# Assessment of Carotid Intima-Media Thickness and Cardiovascular Risk Scores in Bipolar Disorder: A Preliminary Study

**DOI:** 10.1192/j.eurpsy.2026.11212

**Published:** 2026-07-31

**Authors:** Ö. Bayirli, R. Tekdemir, M. T. Ergün, M. AYDIN, H. Özalp, K. M. Gürses, M. U. Yalçın

**Affiliations:** 1 Psychiatry, Selçuk University Medical Faculty; 2 Psychiatry, Meram State Hospital; 3 Cardiology, Selçuk University Medical Faculty, Konya, Türkiye

## Abstract

**Introduction:**

The risk of cardiovascular disease in patients with bipolar disorder is significantly higher than in the general population. This increase is thought to be related to biological processes such as metabolic disorders and vascular changes. Carotid intima-media thickness (CIMT) is a reliable marker of subclinical atherosclerosis. Investigating its relationship with cardiovascular risk scores may help determine the predictive validity of these models in bipolar disorder.

**Objectives:**

This study aims to examine the relationship between CIMT and cardiovascular risk scores (QRisk3, PrimroseBMI, PrimroseLipid, Framingham) in euthymic patients with bipolar disorder.

**Methods:**

The study included 45 euthymic patients diagnosed with bipolar disorder and 25 age- and sex-matched healthy controls. All participants were evaluated using the Structured Clinical Interview for DSM-5 Disorders-Clinician Version (SCID). Cardiovascular risk scores were calculated using the QRisk3, PrimroseBMI, PrimroseLipid, and Framingham models. CIMT was measured using carotid ultrasonography, and the mean value was calculated by averaging the near and far wall intima thicknesses of both common carotid arteries. Ethical approval for the study was obtained from the Selçuk University Local Ethics Committee.

**Results:**

There were no significant differences between the bipolar disorder and healthy control groups in terms of age and sex. Body mass index (BMI) and CIMT were significantly higher in the bipolar disorder group (p < 0.001). QRisk3 and PrimroseLipid scores indicated a higher cardiovascular risk among bipolar patients (p = 0.005 and p = 0.038, respectively). In the bipolar disorder group, CIMT was significantly correlated only with the Framingham score (r = 0.437, p = 0.003). Cardiovascular risk scores showed significant positive correlations with each other (p < 0.001). Patients using lithium had significantly lower CIMT values compared to those not receiving lithium treatment (p = 0.041), whereas no significant differences were found between these groups in terms of cardiovascular risk scores.

**Conclusions:**

Euthymic patients with bipolar disorder show increased CIMT and higher cardiovascular risk compared to controls. Among evaluated models, the Framingham score best reflected subclinical atherosclerosis. The lower CIMT in lithium users suggests a potential protective vascular effect of lithium. Longitudinal studies are needed to confirm these findings.

**Disclosure of Interest:**

None Declared

